# Prediction of Intra-Species Protein-Protein Interactions in Enteropathogens Facilitating Systems Biology Study

**DOI:** 10.1371/journal.pone.0145648

**Published:** 2015-12-30

**Authors:** Ranjan Kumar Barman, Tanmoy Jana, Santasabuj Das, Sudipto Saha

**Affiliations:** 1 Biomedical Informatics Centre, National Institute of Cholera and Enteric Diseases, Kolkata, West Bengal, India; 2 Bioinformatics Centre, Bose Institute, Kolkata, West Bengal, India; 3 Division of Clinical Medicine, National Institute of Cholera and Enteric Diseases, Kolkata, West Bengal, India; National Institute of Genomic Medicine, MEXICO

## Abstract

Protein-protein interactions in *Escherichia coli (E*. *coli)* has been studied extensively using high throughput methods such as tandem affinity purification followed by mass spectrometry and yeast two-hybrid method. This can in turn be used to understand the mechanisms of bacterial cellular processes. However, experimental characterization of such huge amount of interactions data is not available for other important enteropathogens. Here, we propose a support vector machine (SVM)-based prediction model using the known PPIs data of *E*. *coli* that can be used to predict PPIs in other enteropathogens, such as *Vibrio cholerae*, *Salmonella* Typhi, *Shigella flexneri* and *Yersinia entrocolitica*. Different features such as domain-domain association (DDA), network topology, and sequence information were used in developing the SVM model. The proposed model using DDA, degree and amino acid composition features has achieved an accuracy of 82% and 62% on 5-fold cross validation and blind *E*. *coli* datasets, respectively. The predicted interactions were validated by Gene Ontology (GO) semantic similarity measure and String PPIs database (experimental PPIs only). Finally, we have developed a user-friendly webserver named EnPPIpred to predict intra-species PPIs in enteropathogens, which will be of great help for the experimental biologists. The webserver EnPPIpred is freely available at http://bicresources.jcbose.ac.in/ssaha4/EnPPIpred/.

## Introduction

Enteropathogenic bacteria cause deadly diseases such as diarrhea, thyphoid and cholera resulting in high mortality rate, mostly among the children below 5 years [1,2]. The role of proteins and their interactions may provide insight on the mechanism of pathogenesis of the enteropathogenic bacteria. While the PPIs in some bacteria such as *Escherichia coli* (*E*. *coli*) has been well studied through different experimental approaches such as tandem affinity purification followed by mass spectrometry (AP-MS) and yeast two-hybrid (Y2H), it remains unavailable in other pathogens such as *Vibrio cholera* (*V*. *cholera*), *Salmonella* Typhi (*S*. Typhi), *Shigella flexneri* (*S*. *flexneri*) and *Yersinia entrocolitica* (*Y*. *enterocolitica*) [3–6]. The limitations of experimental approaches can be complemented by computational methods for predicting protein interactions [7,8]. While some of the computational approaches are based on sequence coevolution, others use the co-occurrence of interacting protein domains and presence of structural motifs for understanding PPIs [8]. In addition to these, there are machine learning methods to predict the interactions between proteins [9–11]. Chou et al. used gene ontology and pseudo-amino acid composition (GO-PseAA) to predict PPIs of yeast [9]. They obtained overall accuracy of 81.6%, using jackknife cross-validation technique. Similarly, Guo et al. predicted PPIs of yeast with an accuracy of 88.09% using SVM and auto covariance features of protein sequence [10]. Shen et al. employed SVM with a kernel functions and conjoint triad feature of protein sequences to predict PPIs in human [11]. Their best model achieved an average accuracy of 83.9%. In addition, Rashid et al. presented a SVM based method to predict PPIs in *E*. *coli*, *Saccharomyces cerevisiae*, *Helicobacter pylori* using several types of sequence compositions including amino acid, dipeptide, biochemical property, split amino acid and pseudo amino acid composition [12]. In their study, dipeptide composition feature based model achieved an average accuracy of 99.9%, 75.7%, 86.8% in *E*. *coli*, *S*. *cerevisiae* and *H*. *pylori* respectively. Likewise, Guo et al. predicted PPIs in human, yeast, *Drosophila*, *E*. *coli*, and *Caenorhabditis elegans* using SVM [13]. Their methods yield an average accuracy of 90.67%, 88.99%, 90.09%, 92.73%, and 97.51% respectively.

Though all of these prediction methods have achieved a high accuracy in different organisms, there is still unavailability of computational approach where one can predict the PPIs in enteropathogenic bacteria such as *V*. *cholera*, *S*. Typhi, *S*. *flexneri* and *Y*. *enterocolitica*. Here, we present a SVM classifier with 5-fold cross validation techniques, to build an optimal model for known *E*. *coli* PPIs. We extend the optimal SVM model for known *E*. *coli* PPIs to predict PPIs of above mentioned bacteria. We also validate our proposed SVM model by using solved X-ray crystal structures of *E*. *coli* proteins deposited in the Protein Data Bank (PDB) [14]. Predicted PPIs of *S*. Typhi were also validated by Gene Ontology (GO) semantic similarity measure and String PPIs database (Experimental PPIs only) [15]. We developed a webserver EnPPIpred, which allows users to choose different organisms, methods and threshold values to predict enteropathogenic PPIs. Furthermore, users can select one or more pair of proteins available in Swiss-Prot from browse page.

## Materials and Methods

### 2.1 Collection of data

#### 2.1.1 Cross-validation data

*E*. *coli* protein-protein interaction data were downloaded from the Bacterial Protein Interaction Database (Bacteriome.org): integrating physical and functional interactions within the context of an *E*. *coli* knowledgebase [16]. Out of four given datasets, only the TAP interaction dataset by Hu et al (AKA 'Core—experimental'), which was derived from an on-going series of TAP-tag pull-down experiments was used in the present study. 3,886 interactions between 918 proteins were given as a binary interaction format in the dataset. Ensemble Genome ID of all the 918 proteins were collected and converted into Uniprot ID using ID mapping tool. Out of 918 proteins, 912 were successfully mapped, which consists of 3,877 interactions. Out of the above 912 proteins, we got “InterPro” domain hit for 902 proteins. We used the interactions, which had taken place only among the proteins having “InterPro” domain hit to make the positive dataset. Finally, we acquired 3,856 positive interactions among 902 proteins of *E*. *coli*.

As suggested by Ben-Hur et al., a simple uniform random choice of non-interacting protein pairs produce an unbiased estimate of true distribution while predicting protein-protein interactions [17]. Therefore, we chose random protein pairs, which were not found in *E*. *coli* positive dataset as a negative dataset I (positive:negative = 1:1) and negative dataset II (positive:negative-1:10).

#### 2.1.2 Blind dataset

337 positive *E*. *coli* PPIs observed in solved X-ray crystal structures were obtained from the PDB and treated as a blind positive dataset [6]. Therefore, 337 negative protein pairs of *E*. *coli* were generated by random pairing between proteins and treated as a blind negative dataset.

All the datasets used in this study are available in about page of EnPPIpred (http://bicresources.jcbose.ac.in/ssaha4/EnPPIpred/about.php/).

#### 2.1.3 Enteropathogen-independent datasets

All the reviewed proteins of *Salmonella* Typhi (1333), *Vibrio cholerae* (1903), *Shigella flexneri* (2429) and *Yersinia entrocolitica* (857) were downloaded from Protein Knowledgebase, UniProtKB (http://www.uniprot.org/) (dated 15^th^ July, 2014) [18]. In order to predict unknown protein pairs, all the possible protein pairs of above mentioned enteropathogen were generated, and the features vectors were computed.

### 2.2 Feature Vectors

It was shown previously that domain-domain association plays an important role in protein-protein interactions [19]. Considering that we had used domain-domain association as the first feature, Maximum Degree (maximum no. of interacting partner) among protein pairs were considered as the second feature. Degrees of all the 902 *E*. *coli* proteins were calculated from the *E*. *coli* positive dataset. Following Roy et al., we used amino acid composition (AAC) of a protein pair as the next forty [20+20] features of the feature vector [20]. Finally, dipeptide composition (DC) of protein pairs was considered as the next eight hundred features [20*20 + 20*20] of features vectors.

#### 2.2.1. Computation of domain-domain association

Protein related “InterPro” domains were collected from Protein Knowledgebase, UniProtKB. We computed occurrence of particular domain pairs, using similar approach proposed by Sprinzak et al. [21]. We calculated average domain-domain association score (ADDAS) using following equation:

$$\begin{array}{l} ADDAS\;of\;protein\;pair\;(P_{m},P_{n})\;=\sum_{mn=1}^{N}\frac{F_{mn}}{N} \end{array}$$ (1)

where, *F*_*mn*_ is the frequency i.e. no. of protein pairs that contain the domain pair (*D*_*m*_,*D*_*n*_)

*N* is the all possible combination of domain pairs of protein pair (*p*_*m*_, *p*_*n*_).

#### 2.2.2 Amino acid composition

Amino acid composition of all 20 amino acids was calculated as the percentage of each amino acid present in a protein and was calculated using the following equation:

$$Percentage\;of\;amino\;acid\;i=\;\frac{total\;no.\;of\;amino\;acids\left(i\right)}{total\;no.\;of\;amino\;acids\;in\;proteins}\times 100\%$$ (2)

[where *i* can be any natural amino acid]

#### 2.2.3 Dipeptide composition

This encapsulates the information about amino acid composition along with their local order of amino acid. The percentage of each dipeptide was calculated by using Eq 3:

$$\begin{array}{l} Percentage\;of\;dipeptide\;i=\frac{Total\;no.\;of\;dipeptide\;\left(i\right)\;in\;a\;protein}{Total\;no.\;of\;all\;possible\;dipeptides}\times 100\% \end{array}$$ (3)

where *i* denote any dipeptide out of 400 dipeptides.

### 2.3 Support Vector Machines

Support Vector Machines (SVM) was used to classify the interacting and non-interacting protein pairs by greedy search algorithm. We have used SVM^light^ tool provided by T. Joachims [22].

### 2.4 Performance measures

5-fold cross-validation and performance measures including sensitivity, specificity, accuracy, precision (PPV), Mathew’s correlation coefficient (MCC), F1 score and area under ROC curve (AUC) were computed as described in [23].

### 2.5 Semantic similarity measure

The functional properties of two proteins can be estimated by semantic similarity of Gene Ontology (GO) terms. GO covers three important properties of proteins: cellular component (CC), molecular function (MF) and biological process (BP). The ontology is a structure of directed acyclic graph (DAG). All the *S*. Typhi proteins related GO terms were collected from UniProtKB. GO term analysis tools from G-SESAME web server was used to calculate Resnik’s semantic similarity of two GO term sets [24]. Finally the average GO semantic similarity score of all possible combinations of GO terms for an individual protein pair was computed using output GO semantic similarity scores matrix of G-SESAME web server.

### 2.6 STRING

This database contains experimental and predicted PPIs of many organisms. It was used to compare the predicted interactions of SVM based method. In our analysis, we used only experiments checkbox with high confidence score of 0.7, while explicitly excluding the predicted PPIs in STRING.

## Results and Discussion

### 3.1 Selection of features

Different features were tested with the intention to achieve optimal accuracy with nearly equal sensitivity and specificity. As shown in Table A in S1 File, there is no single subset of features that performs better among all. Therefore, we selected a combination of features such as DDA, degree and AAC (default hybrid) features for predicting unknown PPIs of *E*. *coli*, which yields a sensitivity of 77%, specificity of 86% and accuracy of 82% on known *E*. *coli* dataset at a threshold value of 0.00. Since there are no experimentally validated resources for intra-species PPIs for other enteropathogens, we selected DDA and AAC (default hybrid) features for predicting unknown PPIs of others enteropathogens and used in webserver application that achieved an accuracy of 78% with sensitivity of 70% and specificity of 87% on known *E*. *coli* dataset. It was already shown by Memisevic et al. and Roy et al. that DDA and AAC were powerful features for predicting PPIs [19],[20]. Furthermore, we performed 10-fold cross-validation on best subset of features (DDA, Degree and AAC) and compared the result with 5-fold cross-validation. We observed at an optimum threshold value (0.00) the performance measures were similar. However, at extreme threshold values (+1, -1) there are slight variation (Tables A and B in S1 File).

**Fig 1:**
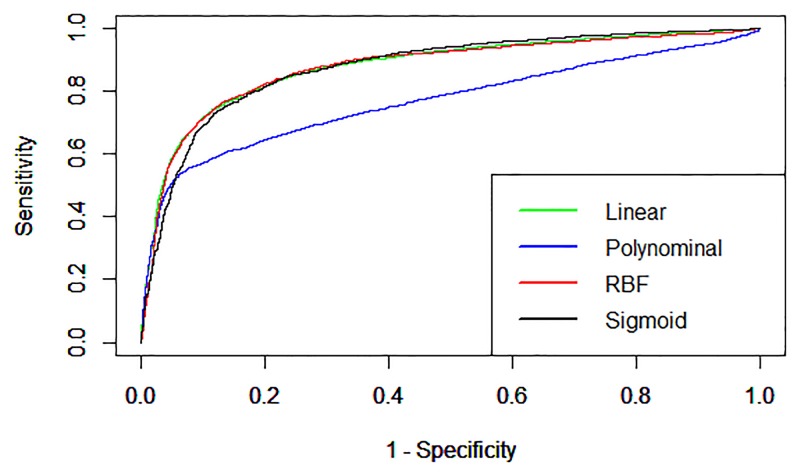
The ROC curves showing performance of different kernels of SVM on *E*. *coli* dataset using optimal subset of features (DDA, degree and AAC).

Different parameters were tested in order to optimize the SVM performance (Table 1). As shown in Fig 1 and Table C in S1 File, the polynomial kernel (parameters t = 1, c = 0.1 and j = 1) showed high specificity (96%), but low sensitivity (49%). In contrast, the linear (parameters t = 0, c = 0.1 and j = 1), radial basis function (RBF) (parameters t = 2, g = 0.001, c = 0.9 and j = 1) and sigmoid (parameters t = 3, s = 0.00001, r = 0.001, c = 1 and j = 3) kernels performed well in terms of sensitivity, specificity and accuracy (73%, 89%, 81%; 77%, 86%, 82% and 80%, 82%, 81% respectively) on *E*. *coli* dataset using optimal subset of features (DDA, degree and AAC). The results indicate that the linear, RBF and sigmoid kernels of SVM performs well rather polynomial. The RBF kernel performs slightly better than linear and sigmoid in terms of accuracy, MCC and F1 score. Therefore we choose RBF model for further analysis.

**Table 1:** SVM performance measures on different subsets of features in *E*. *coli* dataset, using RBF kernel. Optimal parameters were used for respective subset of features.

Features of Protein Pair	Features Vector Length	Sensitivity (%)	Specificity (%)	Accuracy (%)	PPV (%)	MCC	F1 Score (%)	Area under ROC curve
Domain-Domain Association (DDA)	1	32	92	62	80	0.57	45.71	0.633
Degree	1	83	79	81	80	0.62	81.47	0.874
DDA and Degree	2	80	82	81	81	0.62	80.50	0.874
Amino acid composition (AAC)	40	78	76	77	77	0.55	77.50	0.824
DDA and AAC	41	70	87	78	84	0.57	76.36	0.844
Degree and AAC	41	77	85	81	84	0.63	80.35	0.878
**DDA, Degree and AAC**	**42**	**77**	**86**	**82**	**85**	**0.64**	**80.80**	**0.878**
Dipeptide Composition (DC)	800	79	76	77	77	0.55	77.99	0.826
Degree and DC	801	83	79	81	80	0.62	81.47	0.868
DDA, Degree and DC	802	76	86	81	85	0.63	80.25	0.868
DDA, AAC and DC	841	71	88	79	85	0.60	77.37	0.849
All Features (DDA, Degree, AAC and DC)	842	76	87	82	85	0.63	80.25	0.882

All the performance measures were carried out in balance dataset, where size of the negative dataset is equal to the positive dataset (3,856 protein pairs in each case). In addition to these, we tested the performance in imbalanced dataset that closely resemble real-world scenarios, where size of negative dataset is 10 times more than positive dataset (3,856 protein pairs for positive dataset and 38,560 protein pairs for negative dataset). The balance dataset achieved an AUC of 0.878, whereas the imbalance dataset achieved an AUC of 0.861 (Fig A in S1 File).

### 3.2 Performance on blind dataset

The performance of our proposed method was estimated on blind dataset, not used in the training and testing. As shown in Table D in S1 File, at threshold values of -1.00, -1.06 and -1.20, our proposed method attained an accuracy of 63%, 62% and 56%, respectively on the blind set that was collected from the PDB. At these threshold values, the proposed method achieved an accuracy of 76%, 67% and 51%, respectively on 5-fold cross validation of *E*. *coli* dataset (Table A in S1 File).

### 3.3 Validation of enteropathogen-independent dataset

**Fig 2:**
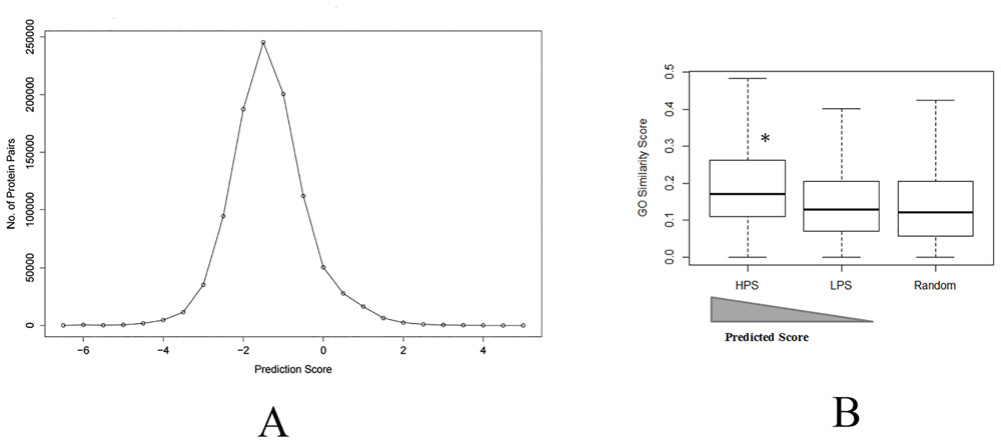
**A**. Frequency plot of *Salmonella* Typhi protein pairs predicted scores. **B**. Box plots represent distribution of GO semantic similarity score vs. SVM prediction score. The x-axis and y-axis of box plots denotes the predicted scores and GO semantic similarity scores respectively. The bins HPS, LPS and Random represents highly predicted scores (Predicted score > 2), low predicted scores (Predicted score ~ -1.5) and random predicted scores (2.73 > Predicted score > -6.33) respectively. Median of each distribution is indicated by a horizontal bar in each box plot. The HPS has significantly (*) higher semantic similarity than LPS (Welch Two Sample t-test P value is 2.2 × 10^−16^) and Random (Welch Two Sample t-test P value is 2.2 × 10^−16^).

All the possible combinations of reviewed protein pairs of *S*. Typhi (1776889 protein pairs), *V*. *cholerae* (3621409 protein pairs), *S*. *flexneri* (5900041 protein pairs) and *Y*.*entrocolitica* (734449 protein pairs) were predicted by the above proposed method. Subsequently, all the predicted scores of protein pairs were plotted using histogram frequency plot function of R. We identified from the frequency plots (as shown in Fig 2A and Figs B-M in S1 File for distribution of protein pairs against prediction scores) of enteropathogen PPIs SVM prediction scores, that tails of all the distribution plots approach the right x-axis at prediction score of 2, but never touch the axis. The prediction scores falling in this area under the curve comprise of very little PPIs of high confidence. Therefore, the threshold value of 2 will give high prediction confidence of PPIs for independent datasets.

**Fig 3:**
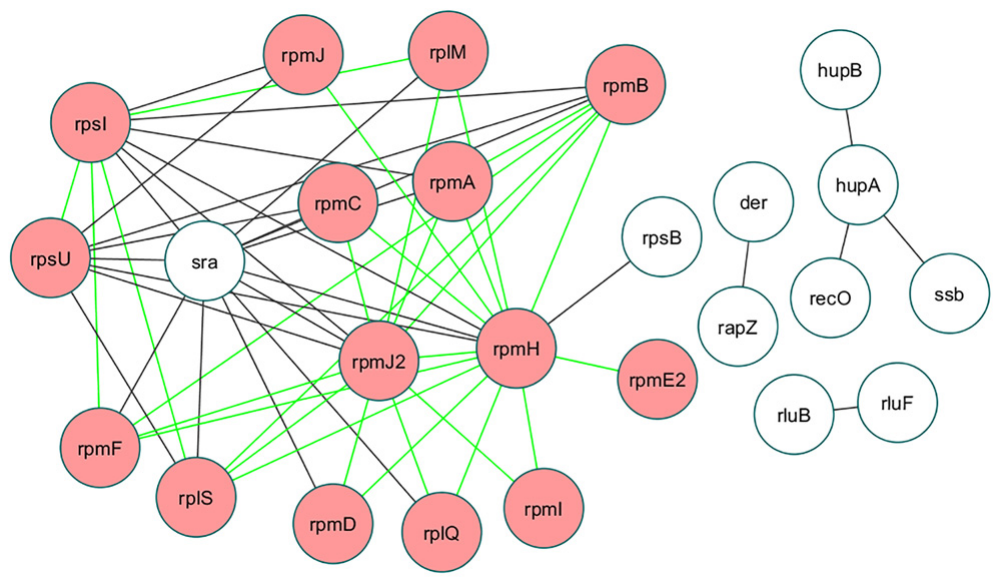
A network of *S*. Typhi protein-protein interactions predicted by our method, where prediction score > 2 and average GO semantic similarity score > 0.7. The network was construct by Cytoscape 3.2.1 [25]. Each node of the network represents *S*. Typhi protein. The green edges of network represent the validated protein-protein interactions of *S*. Typhi by STRING database, where confidence score > 0.7 [15]. Interacting proteins of STRING database (experimental PPIs only) were denoted by pink nodes.

We used GO semantic similarity measure to validate high confidence predicted PPIs of *S*. Typhi. Based on distribution plots, we found that 3357 protein pairs of *S*. Typhi have a prediction score greater than 2 and they were termed as high predicted score (HPS) bin (GO terms of 2968 pairs were observed in HPS). We also took 3357 protein pairs of *S*. Typhi, where prediction score was close to—1.5 and these were treated as a low predicted score (LPS) bin (GO terms of 2733 pairs were observed in LPS). Finally we randomly selected 3357 protein pairs from a total of 1776889 protein pairs and found their corresponding prediction score in between -6.33 to 2.73. We treated these random 3357 protein pairs of *S*. Typhi as a Random bin (GO terms of 2570 pairs were observed in Random set). The Random set may overlap with HPS and LPS, but chances are very low. In our case, we found 6 instances out of 3357, that overlap with HPS but not a single match with LPS set. The distribution of average GO similarity scores in HPS, LPS and Random bins are shown in box plot (Fig 2B). The HPS has significantly (*) higher semantic similarity than LPS (Welch Two Sample t-test P value is 2.2 × 10^−16^) and Random (Welch Two Sample t-test P value is 2.2 × 10^−16^). In addition, the frequency plot of average GO semantic similarity score of HPS was shifted towards right (higher value) as compared to Random set as shown in Fig N in S1 File. These results indicate that, HPS has more accurate interacting protein pairs rather LPS and Random set. A network of *S*. Typhi PPIs (Prediction score > 2 and average GO semantic similarity score > 0.7) is shown in Fig 3. Most of the proteins belong to the ribosomal proteins and many of these predicted interactions are also observed (shown as green edges in Fig 3) in STRING database (experimental PPIs only).

### 3.4 Webserver

**Fig 4:**
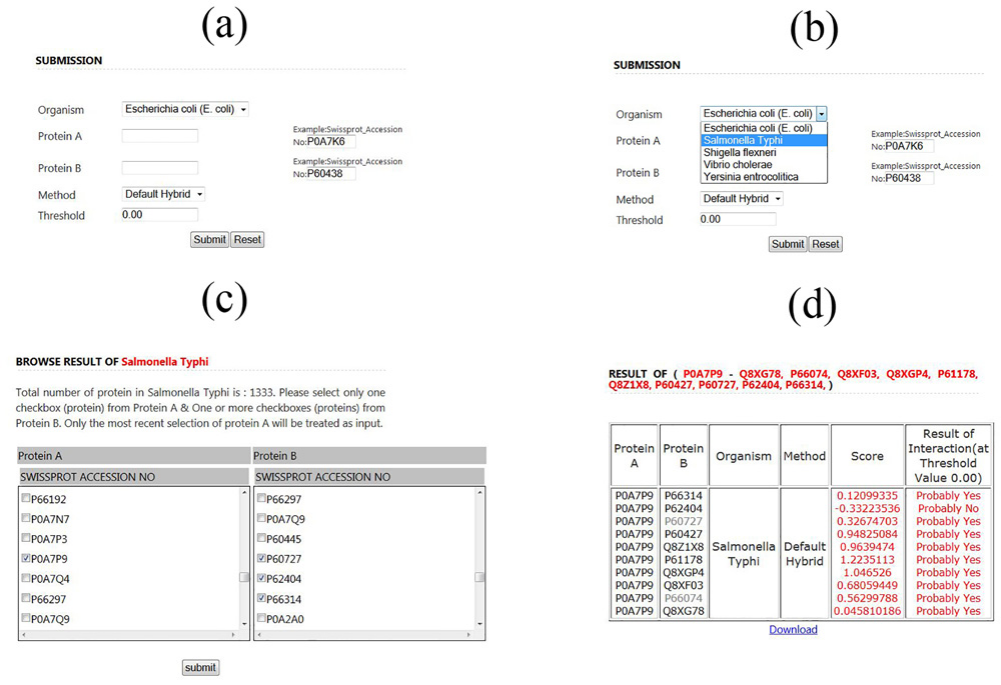
The submission, browse and result pages of EnPPIpred. (a) Submission page, that allows users to select different organisms, features based methods as well as different threshold values. (b) Users can choose different organisms from the dropdown menu. (c) The browse page allows users to choose one from protein A and one or more from protein B, for all the five enteropathogen species. (d) Result page summarizes the output in a tabular format with a prediction score (confidence of prediction) and decision of probable interaction at a given threshold value (default 0.00). Users can get UniProt details about Protein A and B by clicking their UniProtKB AC number in result page.

The web server EnPPIpred was developed to predict intra-species PPIs in enteropathogens. The webserver was developed using PHP and R programing languages, it is available at http://bicresources.jcbose.ac.in/ssaha4/EnPPIpred/. As shown in Fig 4(a), users need to submit two proteins in UniProtKB (Swiss-Prot) AC number format as the input. Users can also select different organisms (Fig 4(b)), methods and threshold values. The Browse option allows users to choose one or more pair of proteins (Protein A and Protein B) available in Swiss-Prot from all the five enteropathogen species (Fig 4(c)). For a given protein pair, the web-server calculates the prediction score and based on which it can be inferred whether the two proteins interact with each other or not. The above results are offered in a tabular format to the user (Fig 4(d)) for a given threshold value, the default value being 0.00. In addition, users can get the details of Protein A and B by clicking their UniProtKB AC number in another page.

## Conclusion

The current study utilized experimentally validated PPIs data of *E*. *coli* and predicted intra-species PPIs of enteropathogenic bacteria such as *V*. *cholera*, *S*. Typhi, *S*. *flexneri* and *Y*. *enterocolitica*. The basic assumption is that similar domains interact in protein level in *E*. *coli* and also in other enteropathogenic bacteria. There are evolutionary conservation of PPIs among enteropathogens at protein level, since domain, amino acid composition and dipeptide composition were trained in known *E*. *coli* PPIs dataset and was able to predict *S*. Typhi unknown PPIs. It was shown that the sequence based features is simple but has the ability to predict PPIs with reasonable accuracy. We observed that the highly predicted PPIs of *S*. Typhi have higher GO semantic similarity score, which shows that SVM models has the ability to classify positive protein-protein pairs of unknown sets. We hope that the EnPPIpred server will play a vital role in enteropathogen interactomics research and many of the predicted pairs will be experimentally validated.

## Supporting Information

S1 File (DOCX)
