# Supplementary material for: Prediction of Intra-Species Protein-Protein Interactions in Enteropathogens Facilitating Systems Biology Study
Source: PLoS One. 2015 Dec 30;10(12):e0145648. doi: 10.1371/journal.pone.0145648 (PMC4699205; doi:10.1371/journal.pone.0145648)
Supplement: S1 File — (DOCX) [file pone.0145648.s001.docx]

**Prediction of intra-species protein-protein interactions in enteropathogens facilitates system biology study.**

Ranjan Kumar Barman^1^, Tanmoy Jana^2^, Santasabuj Das^1,3*^, Sudipto Saha^2^*

**^1^**Biomedical Informatics Centre, National Institute of Cholera and Enteric Diseases, Kolkata, West Bengal, India; **^2^**Bioinformatics Centre, Bose Institute, Kolkata, West Bengal, India; **^3^**Division of Clinical Medicine, National Institute of Cholera and Enteric Diseases, Kolkata, West Bengal, India.

* Corresponding author

**Supporting Information**

**Supporting Table legends**

**Table A.** Different threshold wise SVM performance measures of *E.coli* dataset using 5-fold cross-validation, parameters t = 2 (RBF kernel), g = 0.001, c = 0.9, j = 1 and best subset of features (DDA, Degree and AAC).

**Table B.** Different threshold wise SVM performance measures of *E.coli* dataset using 10-fold cross-validation, parameters t = 2 (RBF kernel), g = 0.001, c = 0.9, j = 1 and best subset of features (DDA, Degree and AAC).

**Table C.** SVM kernel-wise performance measures of *E.coli* dataset, using 5-fold cross-validation. Optimal parameters and threshold value were used for respective kernel. Optimal subset of features (DDA, degree, and amino acid composition (default hybrid)) was used to evaluate the performances of four different SVM kernels.

**Table D.** Performance measures on blind *E. coli* protein-protein interactions (PPIs) dataset (337 positive set was obtained from the Protein Data Bank (PDB) and 337 negative set was obtained from random pairs), using proposed optimal SVM model.

**Supporting Figure legends**

**Fig A** The ROC curves showing performance on balance and imbalance datasets of *E. coli*, using optimal subset of features (DDA, degree and AAC).

**Fig B** Frequency plot of *Salmonella* Typhi protein pairs predicted scores.

**Figs C-F** Frequency plot of *Vibrio cholerae* protein pairs predicted scores.

**Figs G-L** Frequency plot of *Shigella flexneri* protein pairs predicted scores.

**Fig M** Frequency plot of *Yersinia entrocolitica* protein pairs predicted scores.

**Fig N:** Frequency plot of average GO semantic similarity of HPS and Random S. Typhi PPIs.

**Table 1**

| **Threshold** | **Sensitivity  (%)** | **Specificity  (%)** | **Accuracy  (%)** | **PPV  (%)** | **MCC** | **F1 Score   (%)** |
| --- | --- | --- | --- | --- | --- | --- |
| 2.00 | 0 | 20 | 10 | 20 | 0.01 | 0.00 |
| 1.90 | 0 | 40 | 20 | 30 | 0.01 | 0.00 |
| 1.80 | 0 | 80 | 40 | 73 | 0.02 | 0.00 |
| 1.70 | 0 | 80 | 40 | 63 | 0.02 | 0.00 |
| 1.60 | 1 | 100 | 50 | 84 | 0.04 | 1.98 |
| 1.50 | 2 | 100 | 51 | 90 | 0.08 | 3.91 |
| 1.40 | 3 | 100 | 51 | 91 | 0.11 | 5.81 |
| 1.30 | 6 | 99 | 53 | 91 | 0.16 | 11.26 |
| 1.20 | 13 | 99 | 56 | 93 | 0.24 | 22.81 |
| 1.10 | 28 | 98 | 63 | 93 | 0.36 | 43.04 |
| 1.00 | 47 | 97 | 72 | 93 | 0.50 | 62.44 |
| 0.90 | 57 | 95 | 76 | 92 | 0.57 | 70.39 |
| 0.80 | 62 | 94 | 78 | 91 | 0.59 | 73.75 |
| 0.70 | 66 | 93 | 79 | 90 | 0.61 | 76.15 |
| 0.60 | 69 | 91 | 80 | 89 | 0.62 | 77.73 |
| 0.50 | 71 | 90 | 81 | 88 | 0.62 | 78.59 |
| 0.40 | 73 | 89 | 81 | 87 | 0.63 | 79.39 |
| 0.30 | 74 | 88 | 81 | 87 | 0.63 | 79.98 |
| 0.20 | 75 | 88 | 81 | 86 | 0.63 | 80.12 |
| 0.10 | 76 | 87 | 82 | 85 | 0.64 | 80.25 |
| **0.00** | **77** | **86** | **82** | **85** | **0.64** | **80.80** |
| -0.10 | 78 | 85 | 81 | 84 | 0.63 | 80.89 |
| -0.20 | 79 | 84 | 81 | 83 | 0.63 | 80.95 |
| -0.30 | 80 | 83 | 81 | 82 | 0.62 | 80.99 |
| -0.40 | 81 | 82 | 81 | 82 | 0.63 | 81.50 |
| -0.50 | 82 | 81 | 81 | 81 | 0.62 | 81.50 |
| -0.60 | 83 | 79 | 81 | 80 | 0.62 | 81.47 |
| -0.70 | 84 | 78 | 81 | 79 | 0.62 | 81.42 |
| -0.80 | 86 | 75 | 81 | 78 | 0.61 | 81.80 |
| -0.90 | 88 | 72 | 80 | 76 | 0.60 | 81.56 |
| -1.00 | 91 | 61 | 76 | 70 | 0.55 | 79.13 |
| -1.01 | 92 | 58 | 75 | 69 | 0.53 | 78.86 |
| -1.02 | 92 | 55 | 74 | 67 | 0.51 | 77.53 |
| -1.03 | 92 | 51 | 72 | 66 | 0.48 | 76.86 |
| -1.04 | 93 | 48 | 71 | 64 | 0.46 | 75.82 |
| -1.05 | 94 | 44 | 69 | 63 | 0.43 | 75.44 |
| -1.06 | 95 | 39 | 67 | 61 | 0.40 | 74.29 |
| -1.07 | 95 | 34 | 65 | 59 | 0.37 | 72.79 |
| -1.08 | 96 | 30 | 63 | 58 | 0.34 | 72.31 |
| -1.09 | 97 | 26 | 61 | 56 | 0.32 | 71.01 |
| -1.10 | 97 | 21 | 59 | 55 | 0.28 | 70.20 |
| -1.20 | 99 | 3 | 51 | 51 | 0.09 | 67.32 |
| -1.30 | 100 | 1 | 50 | 50 | 0.06 | 66.67 |
| -1.40 | 100 | 0 | 50 | 50 | 0.03 | 66.67 |
| -1.50 | 100 | 0 | 50 | 50 | 0.01 | 66.67 |
| -1.60 | 100 | 0 | 50 | 50 | 0.00 | 66.67 |
| -1.70 | 100 | 0 | 50 | 50 | 0.00 | 66.67 |
| -1.80 | 100 | 0 | 50 | 50 | 0.00 | 66.67 |
| -1.90 | 100 | 0 | 50 | 50 | 0.00 | 66.67 |
| -2.00 | 100 | 0 | 50 | 50 | 0.00 | 66.67 |

**Table 2**

| **Threshold** | **Sensitivity  (%)** | **Specificity  (%)** | **Accuracy  (%)** | **PPV  (%)** | **MCC** | **F1 Score   (%)** |
| --- | --- | --- | --- | --- | --- | --- |
| 1.00 | 51 | 96 | 74 | 93 | 0.54 | 65.88 |
| 0.90 | 60 | 95 | 78 | 92 | 0.59 | 72.63 |
| 0.80 | 65 | 94 | 79 | 91 | 0.61 | 75.83 |
| 0.70 | 68 | 92 | 80 | 90 | 0.62 | 77.47 |
| 0.60 | 70 | 91 | 81 | 89 | 0.63 | 78.36 |
| 0.50 | 72 | 90 | 81 | 88 | 0.63 | 79.20 |
| 0.40 | 73 | 89 | 81 | 87 | 0.63 | 79.39 |
| 0.30 | 74 | 88 | 81 | 87 | 0.63 | 79.98 |
| 0.20 | 76 | 88 | 82 | 86 | 0.64 | 80.69 |
| 0.10 | 76 | 87 | 82 | 85 | 0.64 | 80.25 |
| **0.00** | **77** | **86** | **82** | **85** | **0.64** | **80.80** |
| -0.10 | 78 | 85 | 81 | 84 | 0.63 | 80.89 |
| -0.20 | 79 | 84 | 81 | 83 | 0.63 | 80.95 |
| -0.30 | 80 | 83 | 81 | 82 | 0.63 | 80.99 |
| -0.40 | 81 | 82 | 81 | 82 | 0.63 | 81.50 |
| -0.50 | 82 | 80 | 81 | 81 | 0.63 | 81.50 |
| -0.60 | 83 | 79 | 81 | 80 | 0.62 | 81.47 |
| -0.70 | 85 | 77 | 81 | 79 | 0.62 | 81.89 |
| -0.80 | 86 | 75 | 80 | 77 | 0.61 | 81.25 |
| -0.90 | 88 | 71 | 80 | 75 | 0.60 | 80.98 |
| -1.00 | 92 | 58 | 75 | 69 | 0.53 | 78.86 |

**Table 3**

| **SVM Kernel** | **Sensitivity**  **(%)** | **Specificity**  **(%)** | **Accuracy**  **(%)** | **PPV**  **(%)** | **MCC** | **F1 Score**  **(%)** | **Area under ROC cure** |
| --- | --- | --- | --- | --- | --- | --- | --- |
| Linear | 73 | 89 | 81 | 87 | 0.63 | 79.39 | 0.881 |
| Polynomial | 49 | 96 | 72 | 92 | 0.51 | 63.94 | 0.769 |
| Radial basis function | **77** | **86** | **82** | **85** | **0.64** | **80.80** | **0.878** |
| Sigmoid | 80 | 82 | 81 | 81 | 0.62 | 80.50 | 0.877 |

**Table 4**

| **Threshold** | **Sensitivity (%)** | **Specificity (%)** | **Accuracy (%)** | **PPV (%)** | **MCC** | **F1 Score (%)** |
| --- | --- | --- | --- | --- | --- | --- |
| 1.00 | 6 | 99 | 47 | 92 | 0.14 | 11.27 |
| 0.90 | 9 | 99 | 49 | 89 | 0.17 | 16.35 |
| 0.80 | 11 | 98 | 49 | 89 | 0.18 | 19.58 |
| 0.70 | 13 | 98 | 50 | 87 | 0.19 | 22.62 |
| 0.60 | 14 | 97 | 51 | 86 | 0.19 | 24.08 |
| 0.50 | 15 | 97 | 51 | 85 | 0.20 | 25.50 |
| 0.40 | 17 | 96 | 52 | 85 | 0.21 | 28.33 |
| 0.30 | 19 | 96 | 53 | 86 | 0.22 | 31.12 |
| 0.20 | 19 | 96 | 53 | 85 | 0.22 | 31.06 |
| 0.10 | 19 | 96 | 53 | 85 | 0.22 | 31.06 |
| 0.00 | 20 | 95 | 53 | 85 | 0.23 | 32.38 |
| -0.10 | 21 | 95 | 53 | 84 | 0.23 | 33.60 |
| -0.20 | 22 | 95 | 54 | 85 | 0.24 | 34.95 |
| -0.30 | 24 | 95 | 55 | 85 | 0.25 | 37.43 |
| -0.40 | 24 | 94 | 55 | 84 | 0.25 | 37.33 |
| -0.50 | 27 | 94 | 56 | 85 | 0.27 | 40.98 |
| -0.60 | 28 | 93 | 57 | 84 | 0.27 | 42.00 |
| -0.70 | 31 | 93 | 58 | 85 | 0.30 | 45.43 |
| -0.80 | 33 | 93 | 60 | 86 | 0.32 | 47.70 |
| -0.90 | 36 | 92 | 61 | 84 | 0.32 | 50.40 |
| -1.00 | 47 | 85 | 63 | 79 | 0.33 | 58.94 |
| -1.01 | 49 | 83 | 64 | 79 | 0.34 | 60.48 |
| -1.02 | 51 | 82 | 65 | 78 | 0.34 | 61.67 |
| -1.03 | 53 | 78 | 64 | 76 | 0.32 | 62.45 |
| -1.04 | 55 | 73 | 63 | 72 | 0.28 | 62.36 |
| -1.05 | 58 | 68 | 62 | 70 | 0.26 | 63.44 |
| **-1.06** | **61** | **64** | **62** | **68** | **0.25** | **64.31** |
| -1.07 | 64 | 59 | 62 | 66 | 0.23 | 64.98 |
| -1.08 | 66 | 49 | 59 | 62 | 0.15 | 63.94 |
| -1.09 | 71 | 42 | 58 | 61 | 0.14 | 65.62 |
| -1.10 | 75 | 38 | 58 | 60 | 0.13 | 66.67 |
| -1.20 | 96 | 4 | 56 | 56 | 0.01 | 70.74 |
| -1.30 | 100 | 0 | 56 | 56 | 0.00 | 71.79 |
| -1.40 | 100 | 0 | 56 | 56 | 0.00 | 71.79 |
| -1.50 | 100 | 0 | 56 | 56 | 0.00 | 71.79 |

**
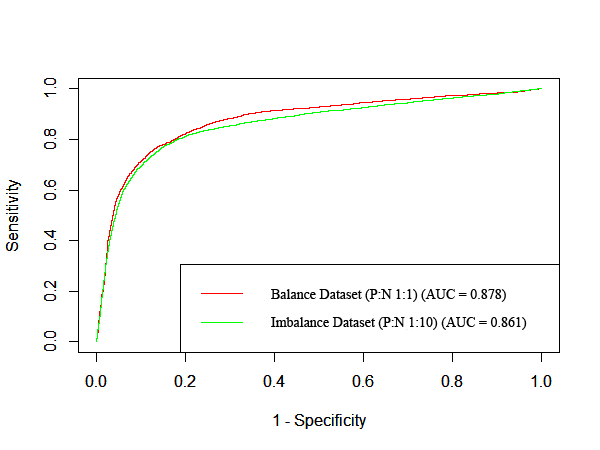
**

**Fig A**

**
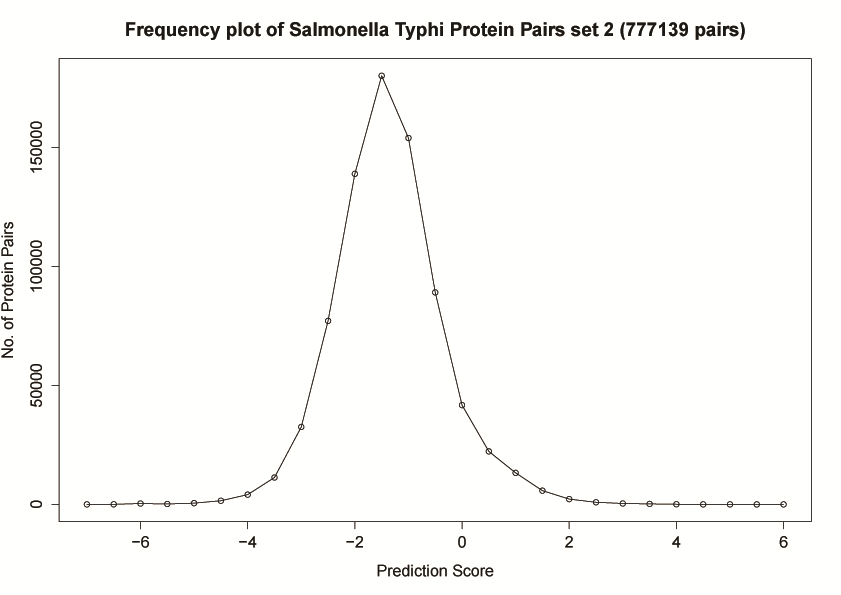
**

**Fig B**

**
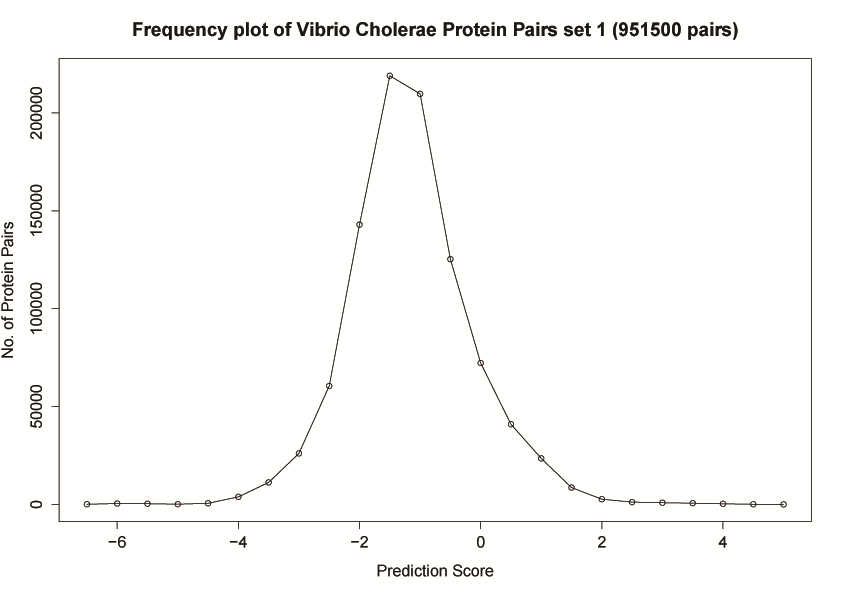
**

**Fig C**

**
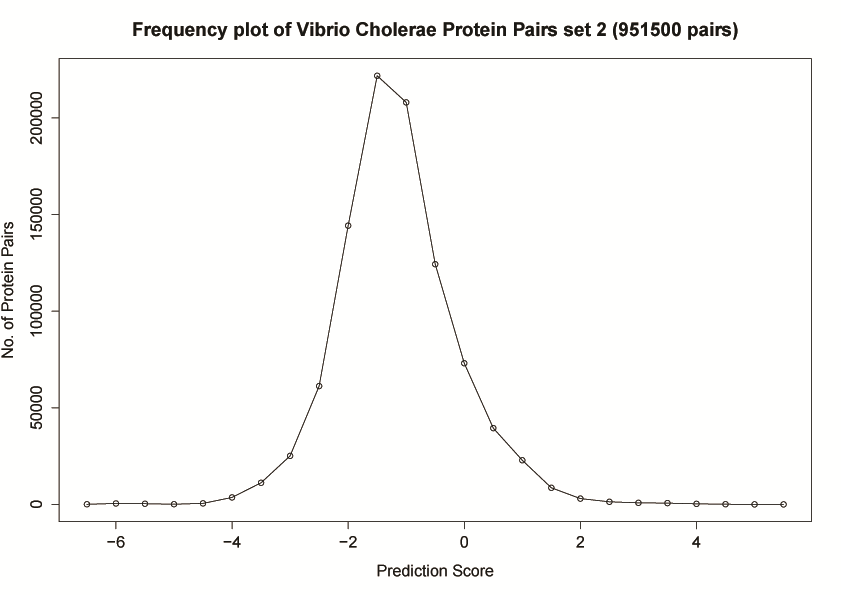
**

**Fig D**


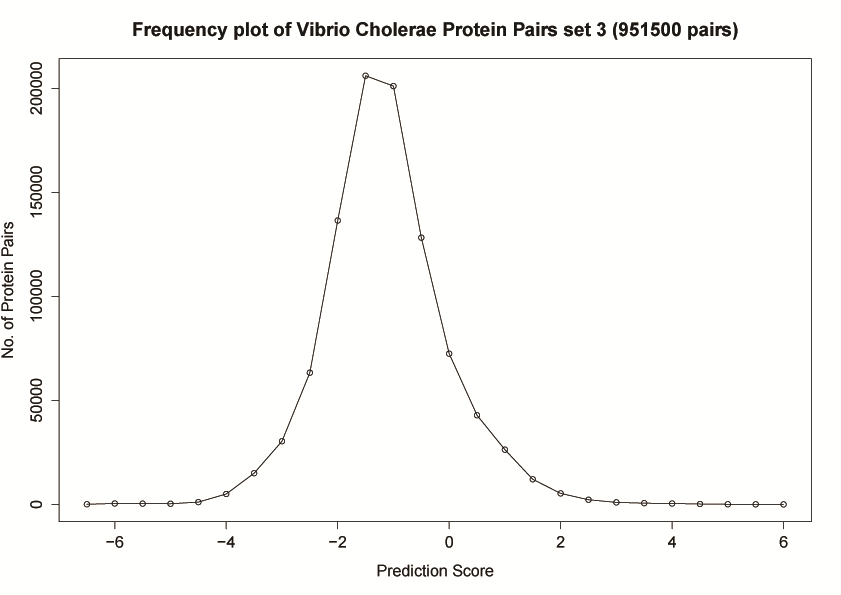


**Fig E**


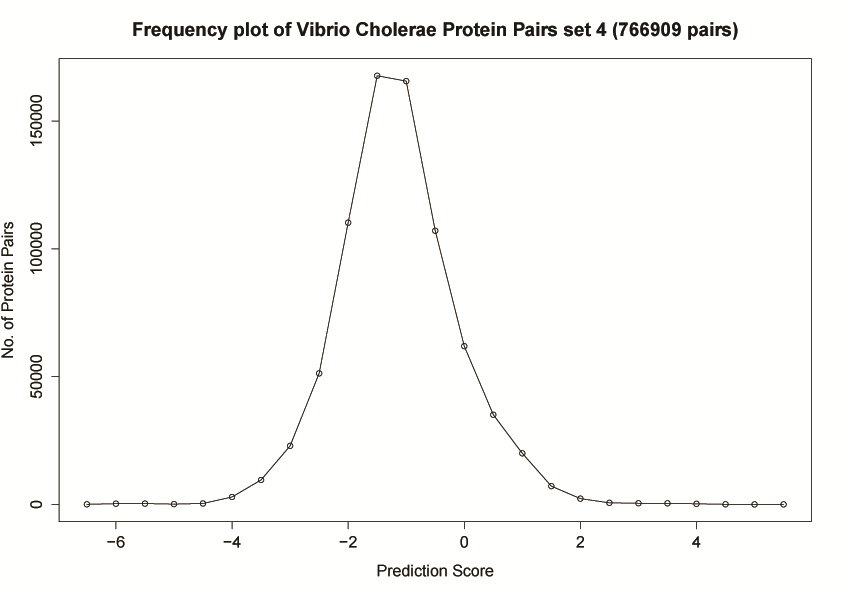


**Fig F**


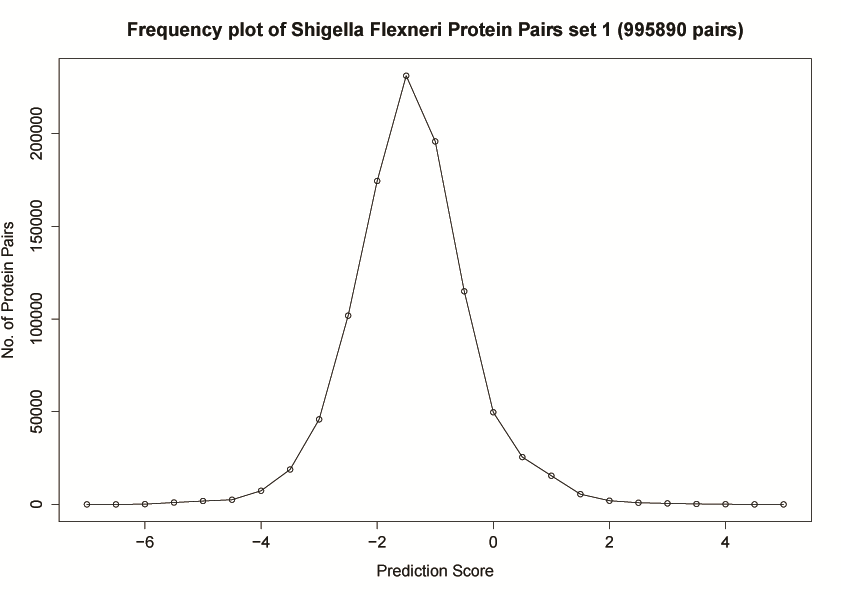


**Fig G**

**
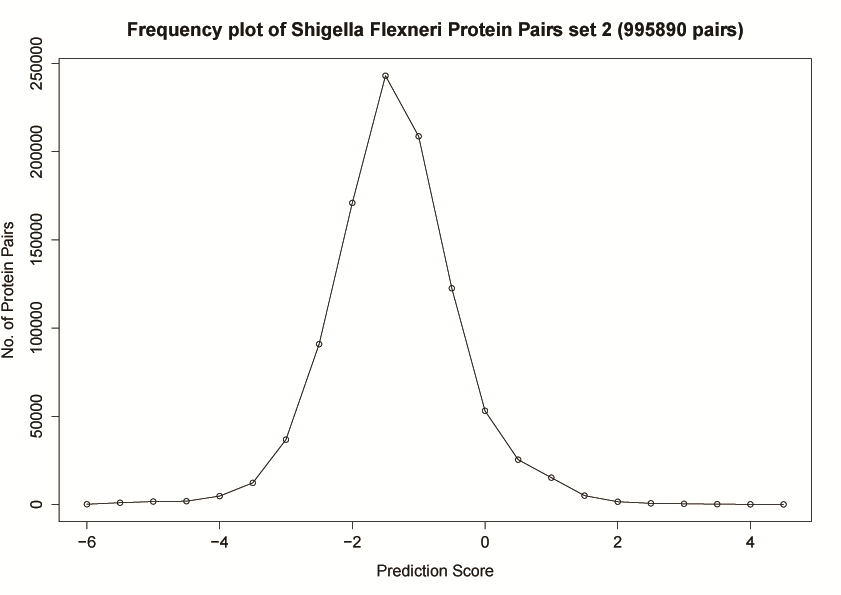
**

**Fig H**

**
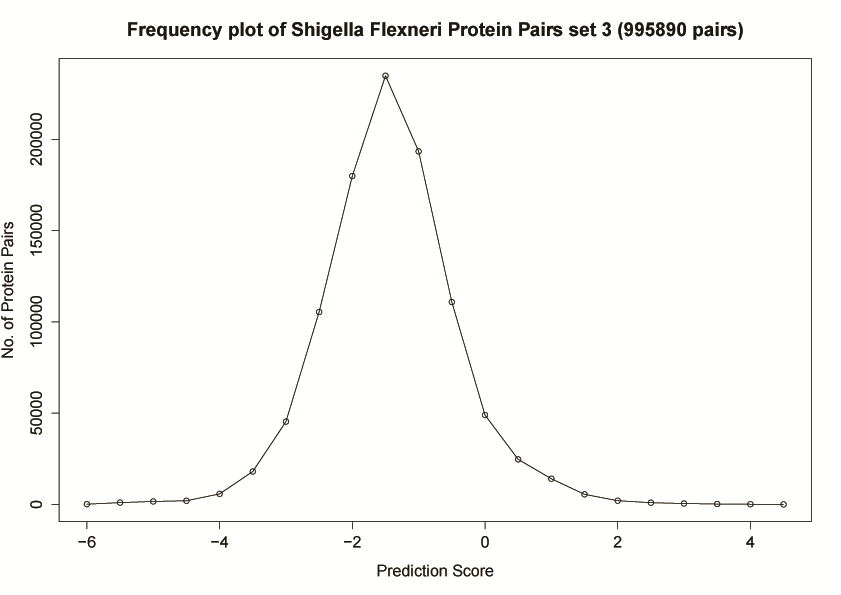
**

**Fig I**

**
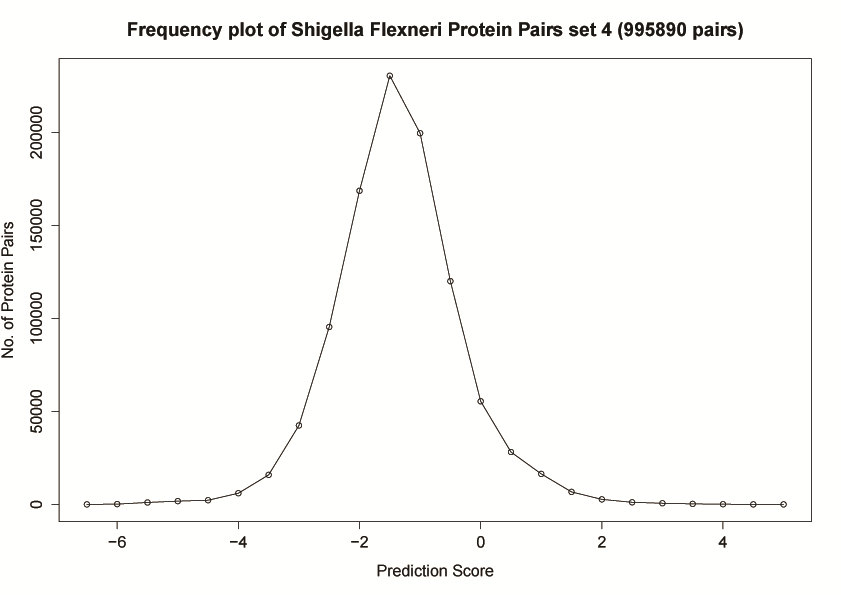
**

**Fig J**

**
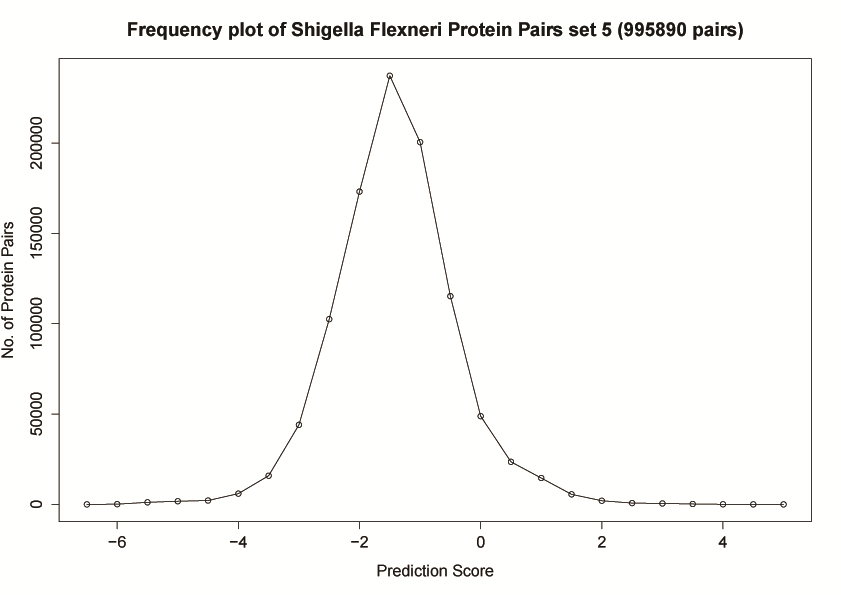
**

**Fig K**

**
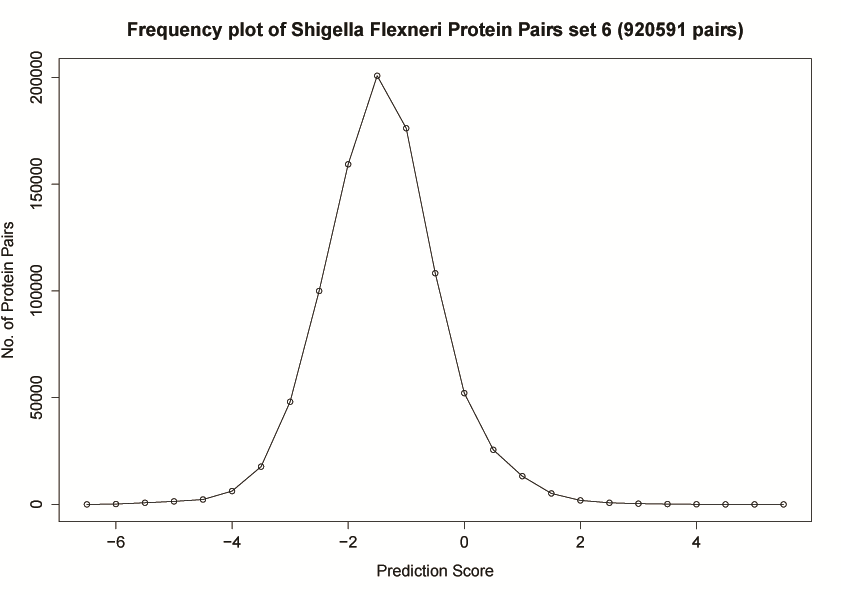
**

**Fig L**

**
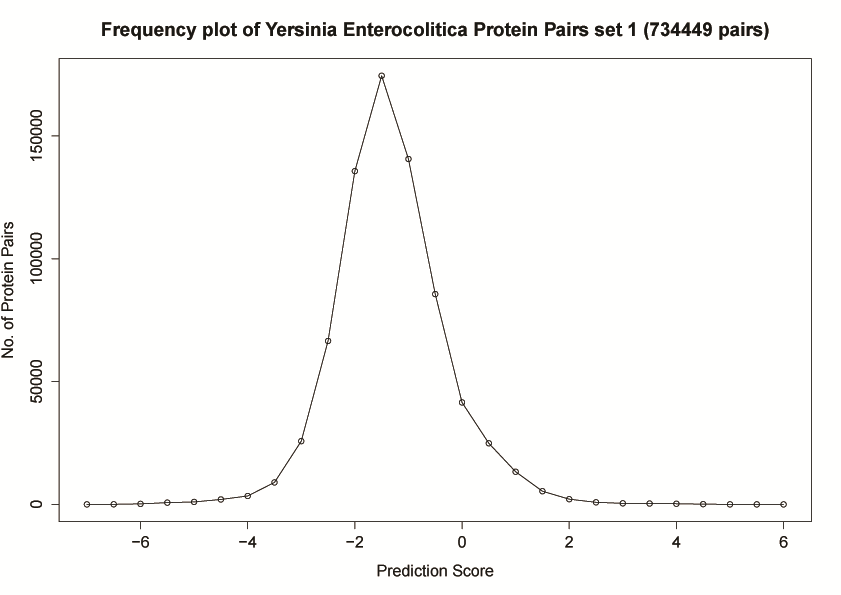
**

**Fig M**


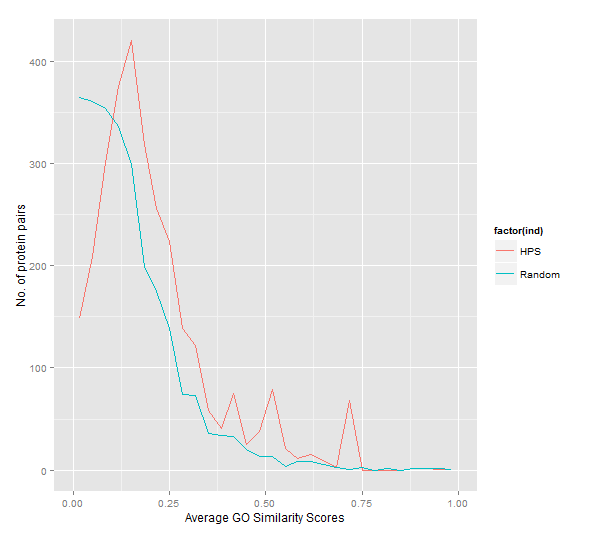


**Fig N**
